# Supporting Youth Participation in Health and Climate Justice Through Advocacy Training: Comment on "Between Rhetoric and Reality: Learnings From Youth Participation in the Adolescent and Youth Health Policy in South Africa"

**DOI:** 10.34172/ijhpm.2023.7898

**Published:** 2023-11-20

**Authors:** L. Katie O'Connell, Nisha Botchwey

**Affiliations:** ^1^Georgia Institute of Technology, School of City and Regional Planning, Atlanta, GA, USA; ^2^Humphrey School of Public Affairs, University of Minnesota, Minneapolis, MN, USA

**Keywords:** Youth Participation, Youth Advocacy, Climate Change, Health Policy

## Abstract

This paper responds to lessons from the Adolescent and Youth Health Policy (AYHP) process in South Africa by drawing comparisons with youth participation within the climate justice movement. Relationship building is essential to successful youth participation in health policy and climate change as it creates intergenerational learning and cross-cultural engagement. At the same time, both sets of youth also deal with compounding challenges due to contemporary and historical legacies of colonialism and inequality. Yet, tokenism challenges the participatory process as adults profess to value youth perspectives, yet recommendations by youth often do not get incorporated into policies or plans. For organizations and agencies trying to build youth’s capacity, organizations and agencies should look to programs that train youth in advocacy. These programs help build youth’s confidence, increase their optimism for change, and give youth a sense of ownership.

 Jacobs and George’s paper^1^ looks to understand the role of youth within health policy-making in South Africa. They do this by studying the “Adolescent and Youth Health Policy” (AYHP) process, which engages youth to work with government agencies, researchers, and civil society to develop health policy. To understand AYHP, Jacobs and George did a qualitative analysis of interviews with policy actors using a conceptual framework rooted in feminist, post-structural, and critical theory. They found that AYHP supported the inclusion of youth within South African health policy-making. Yet, challenges still need to be addressed to ensure the full integration of youth within the process. They see this analysis as part of the broader aim of understanding how to support youth participation within health policy.

 The Jacob and George article is valuable to youth participation literature, especially as it overlays lessons from youth participation in climate justice. Within the climate movement, global youth actions have helped re-envision youth as change agents rather than passive victims of climate disasters. Recognizing the overlap between youth participation in climate and health is essential to foster youth agency, especially as we aim to build a more just and equal world.

 An essential part of creating strong youth participation is building relationships between various policy actors, including government, agencies, researchers, and youth activists. For example, within the climate justice movement, there is the “Youth in Landscapes Initiative,” a subsection of the Global Landscapes Forum. The program brings together youth from around the world for a masterclass on engaged participation in science policy and sustainability. Youth have the opportunity to meet one another as well as senior professionals, creating an opportunity for intergenerational learning between youth, scientists, and policy-makers.^2^ Programs that develop youth-adult relationships not only foster intergenerational connections and facilitate the exchange of knowledge, but they can also enhance communication and negotiations between young people and adults, which is often impeded by generational divides.^3^

 Another program aimed at building relationships, “Youth Leading Environmental Change,” convenes students from six countries (Bangladesh, Canada, Germany, India, Uganda, and the United States) to participate in a workshop series focused on environmental issues, including climate change and environmental justice. The program develops peer role models through cross-cultural engagement and enables youth to see themselves as part of a global movement.^5^ By identifying as part of a global movement, a diverse coalition of youth from various backgrounds, each with unique cognitive abilities and worldviews, can counter the idea that the youth climate movement should echo a singular voice led by a handful of individuals. Instead, the movement gains strength by embracing an array of voices and local experiences which better communicate climate science as a lived reality.^5^

 Developing peer networks are an opportunity to create transnational solidarity,^6^ which is especially vital for marginalized youth, who often sit at the intersection of contemporary and historical inequalities. Jacobs and George describe these overlapping disparities that combine with the embedded legacies of colonialism and apartheid to compound South African youth’s health challenges, similar to youth living in areas most vulnerable to climate change. For example, indigenous populations worldwide who often have tenuous land rights depend most on the land for livelihoods and well-being.^7^ Challenges due to climate disasters include further loss of land and disruptions to long-established relationships with the local ecosystem.

 Youth participating in health policy or climate justice face similar challenges to being recognized by adults as full members of the process. As Jacobs and George note, youth perspectives were not always incorporated into plans, potentially perpetuating a system of tokenism. Likewise, within climate justice, youth want to be seen as capable partners in developing strategies.^8^ Instead, they are seen as learners, not contributors or producers, even at youth-centered events, like the Youth Non-Governmental Organizations meetings for the United Nations Framework Convention on Climate Change.^9^ At the same time, just as AYAP used youth as experts but excluded them during final decision-making, youth climate recommendations can be challenging to incorporate within municipal plans, even within countries that have ratified the United Nations Convention on the Rights of the Child. This can be because local governments do not prioritize youth voices because they are not yet viewed as full members of society. Institutions need systems that give youth a platform for ideas and integrate their recommendations.^10^

 Adults looking to expand the role of youth within health policy and climate justice should think about the participatory process using Arnstein’s metaphorical ladder as a guide. At the base, citizens are non-participants who contend with power holders that rely on manipulation as a form of control. Above nonparticipation is tokenism which includes the three rungs of informing, consultation, and placation. The lack of implementation of ideas follows a classic placation strategy that uses citizen participation as a demonstration of involvement rather than a moment of power sharing. After tokenism, as citizens gain power and become integral to the decision-making process, they move up the ladder until the pinnacle rung, which is a system based on citizen control.^11^

 A youth participation ladder has similar corresponding levels but with a typology of partnership between youth and adults.^12^ Botchwey et al^13^ expanded the youth participation ladder to include new rungs – consent, advocacy, and incorporation. *Consent* is when adults give youth permission to participate, often by giving youth a platform to share their perspectives. Within this rung, youth recommendations must produce adult action, or the process threatens to shift toward tokenism. *Advocacy* allows youth to promote their ideas for system changes, which adults do not solicit. Instead, designs are youth-led. Finally, *incorporation* is when the youth and adults work together, from planning to implementation. At this rung, youth voices must be valued and integrated within institutions.

 Moving from rhetoric to the reality of youth participation requires scaffolding youth’s capacity, which can be done by focusing on youth-led advocacy projects. These action projects support self-efficacy and help youth recognize themselves beyond their personal lives.^4^ For example, the “Youth Engagement and Action for Health” (YEAH!) program engages youth in creating healthy neighborhoods by adult leaders training youth to do neighborhood assessments, followed by the youth selecting a project and advocating their ideas to local decision-makers. After completing the 14-session curriculum, youth participants in YEAH! increased their optimism for change, assertiveness, and ability to influence decisions within a group.^14^ As youth see their actions have an impact, they begin to recognize their power to change situations,^15^ which builds a sense of ownership,^16^ a crucial component for complex issues like health policy or climate justice.

 Whether policy-makers focus their efforts on improving communities’ health conditions or working to thwart climate change, youth must play a meaningful role in the decision-making process. Youth brings a wide breadth of experiences and knowledge that differ from adults, and by ensuring all ages of society participate, a more robust system is created. As Jacobs and George demonstrate with AYHP, authentic youth participation requires strong relationships that value the youth voice and create systems that include youth perspectives. Programs like YEAH!, that train youth to be community advocates, should gain recognition as an essential tool in developing active youth citizens. The current generation of young people distinctly aspire to be catalysts for change in social and environmental justice movements,^17^ a sentiment acknowledged by adult leaders who recognize the importance of youth perspectives and insights. For tangible change to occur, it is imperative for policy-makers and governmental agencies to allocate time and resources to programs that ensure youth are not just participants but incorporated into the decision-making processes.

## Ethical issues

 Not applicable.

## Competing interests

 Authors declare that they have no competing interests.
